# Successful sequential therapy for *Stenotrophomonas maltophilia* infection in a preterm neonate: a case report

**DOI:** 10.3389/fped.2025.1619075

**Published:** 2025-09-04

**Authors:** Majed Nahari, Mohammed Alaboud, Syed Mohinuddin, Maheer Faden, Yasser Balhareth, Noura Alsaleem

**Affiliations:** ^1^ Pharmaceutical Care Services, King Abdullah Bin Abdulaziz University Hospital, Riyadh, Saudi Arabia; ^2^ College of Pharmacy, Princess Nourah Bint Abdulrahman University, Riyadh, Saudi Arabia; ^3^ Children's Health Department, King Abdullah Bin Abdulaziz University Hospital, Riyadh, Saudi Arabia; ^4^ College of Medicine, Princess Nourah Bint Abdulrahman University, Riyadh, Saudi Arabia; ^5^ Department of Pediatrics, Faculty of Medicine, University of Ottawa, Ottawa, ON, Canada; ^6^ Newborn Care, Children's Hospital of Eastern Ontario, Ottawa, ON, Canada; ^7^ NICU Division, Children's Health Department, King Abdullah Bin Abdulaziz University Hospital, Riyadh, Saudi Arabia

**Keywords:** *Stenotrophomonas maltophilia*, neonatal sepsis, multidrug resistance, trimethoprim-sulfamethoxazole, levofloxacin

## Abstract

**Background:**

A multidrug-resistant pathogen increasingly seen in newborn intensive care units, *stenotrophomonas maltophilia* presents treatment difficulties for preterm babies.

**Case presentation:**

A 28-week preterm neonate developed *S. maltophilia* sepsis following prolonged mechanical ventilation. Initial therapy with trimethoprim- sulfamethoxazole (TMP-SMX) was ineffective, prompting a switch to levofloxacin, which led to clinical recovery and infection clearance.

**Conclusion:**

This case highlights the need for tailored antimicrobial strategies in neonatal *S. maltophilia* infections. Sequential therapy with TMP-SMX and levofloxacin was effective, supporting the judicious use of fluoroquinolones in resistant cases.

## Introduction

Preterm neonates face an increased risk of nosocomial infections due to their immunologic immaturity, low birth weight, and frequent exposure to invasive medical procedures such as intravascular catheterization and mechanical ventilation (1). These vulnerabilities lead to the emergence of multidrug-resistant organisms, complicating clinical management and raising morbidity and mortality (2).

*Stenotrophomonas maltophilia* is an opportunistic gram-negative aerobic bacillus that has emerged as a significant nosocomial pathogen, particularly in immunocompromised individuals, including neonates (3). Its intrinsic resistance to multiple classes of antibiotics presents a considerable challenge in clinical practice (4). Infections caused by *S. maltophilia* can impact various organ systems, including the bloodstream, respiratory tract, urinary tract, and central nervous system, with pneumonia and sepsis being the most common presentations in neonates (5).

The management of *S. maltophilia* infections in neonates is further complicated by limited pharmacokinetic data and safety concerns regarding effective antimicrobial agents (5). This case report highlights the successful sequential use of trimethoprim- sulfamethoxazole (TMP-SMX) followed by levofloxacin in the treatment of a preterm neonate with *S. maltophilia* sepsis.

## Case presentation

A female preterm neonate was delivered at 28 weeks and 2 days of gestation via emergency cesarean section, necessitated by maternal antepartum hemorrhage. She was admitted to the neonatal intensive care unit (NICU) because of respiratory distress syndrome, necessitating mechanical ventilation. On the 17th day of life, the newborn exhibited clinical deterioration following a video laryngoscopic evaluation for suspected difficult intubation, probably due to aspiration.

Empirical treatment involving cloxacillin and gentamicin was commenced, yet there was no observed clinical improvement. Meropenem, gentamicin, and vancomycin were incorporated into the antibiotic regimen, leading to a temporary stabilization of the patient. Tracheal aspirate culture revealed the presence of *Stenotrophomonas maltophilia* and Acinetobacter baumannii. After consulting with infectious disease specialists, the decision was made to discontinue vancomycin. Instead, a regimen of ceftazidime along with trimethoprim-sulfamethoxazole (TMP-SMX) was initiated at a dosage of 12 mg/kg every 8 h for a period of 10 days. Prior to initiating TMP- SMX, both total and direct serum bilirubin levels were within normal ranges. Daily monitoring during the first three days, followed by assessments every other day, showed no significant changes.

Even after finishing the TMP-SMX treatment, later tracheal aspirates still showed positive results for *S. maltophilia*, along with increasing oxygen requirements. As a result, levofloxacin was initiated at a dosage of 10 mg/kg twice daily for a duration of 10 days, leading to notable clinical improvement and negative follow-up cultures, thereby confirming the eradication of the infection. Throughout the hospitalization, blood cultures consistently returned negative results. The laboratory findings during TMP-SMX and levofloxacin therapy are summarized in Table 1.

**Table 1:** Laboratory findings during TMP-SMX and levofloxacin therapy.

Day of therapy	Treatment	CRP (mg/L)	WBC (×10⁹/L)	Neutrophils (×10⁹/L)	Platelets (×10⁹/L)	Total bilirubin (µmol/L)
Day 1	TMP-SMX	7.8	9.7	4.8	244	39
Day 2	TMP-SMX	4.5	10.9	5.99	236	33.8
Day 4	TMP-SMX	2.4	14.9	6.0	115	17.6
Day 7	TMP-SMX	5.9	21.6	18.5	274	3.3
Day 9	TMP-SMX	39.6	—	—	—	—
Day 10	TMP-SMX	17.0	—	—	—	—
Day 1	Levofloxacin	5.8	17.3	11.9	260	23
Day 6	Levofloxacin	4.8	14.7	9.8	189	—
Day 10	Levofloxacin	5.8	13.9	9.45	141	13.3

## Discussion

This case highlights the complexities involved in managing S. maltophilia infections in preterm neonates, especially those requiring prolonged respiratory support. The sequential approach, starting with TMP-SMX followed by levofloxacin, was guided by clinical response and microbiological data. Although TMP-SMX is the first-line therapy for S. maltophilia infections, treatment failures are not uncommon, particularly in severe or persistent cases (6).

The intrinsic resistance of S. maltophilia to multiple antibiotics necessitates a tailored, susceptibility-guided therapeutic strategy. While fluoroquinolones like levofloxacin are not typically first-line agents in neonates due to concerns about cartilage toxicity, emerging evidence supports their role in multidrug-resistant (MDR) S. maltophilia infections when conventional treatments fail (7). Our findings are consistent with the recent case series by Verma et al., which reported an 80% success rate among five preterm neonates with S. maltophilia sepsis (8). In their series, only one patient received TMP-SMX alone, while the others were treated with levofloxacin alone (three cases), levofloxacin plus amikacin (one case), or minocycline plus amikacin and TMP-SMX (one case). These findings highlight the variable use of TMP-SMX and the potential role of fluoroquinolones, either alone or in combination, as effective options in challenging cases. The evidence from recent neonatal studies and case reports on treatment efficacy for *S. maltophilia* infections is summarized in Table 2.

**Table 2:** Summarizes the evidence from recent neonatal studies and case reports on treatment efficacy for *S. maltophilia* infections.

#	Study	Year	Study type	Sample size	Infection type	Treatment	Successful (n)	Success rate (%)
1	Ryan et al.	2013	Case report	1	Pneumonia	TMP-SMX	1	100%
2	Newby et al.	2017	Case series	3	MDR infection	Levofloxacin	3	100%
3	Kim et al.	2022	Case report	1	Meningitis	Levofloxacin	1	100%
4	Zhang et al.	2023	Case series	5	Sepsis	TMP-SMX + Fluoroquinolones	3	60%
5	Shao et al.	2023	Retrospective	22	VAP	TMP-SMX, ceftazidime	14	63.6%
6	Adefila et al.	2024	Case report	2	Sepsis	TMP-SMX	1	50%
7	Trauth et al.	2024	Case report	1	Nosocomial Pneumonia	Cefiderocol	1	100%
8	Verma et al.	2024	Case series	5	Sepsis	(TMP-SMX alone, Levofloxacin alone, Levofloxacin + Amikacin, Minocycline + Amikacin + TMX-SMX)	4	80%

Emerging literature demonstrates the successful use of alternative therapies in neonatal S. maltophilia infections. Adefila et al. reported a case successfully treated with a tailored antimicrobial approach guided by local antibiogram data (9). Similarly, cefiderocol has been explored as a novel treatment strategy, although limited clinical data and availability constraints may restrict its widespread use in NICUs (10).

Although the pharmacokinetics of levofloxacin in neonates have not been extensively studied, available data suggest adequate tissue penetration and bactericidal activity against S. maltophilia. In this case, levofloxacin was well-tolerated, with no immediate or delayed adverse effects observed. The cautious use of fluoroquinolones and close monitoring for potential side effects remains essential in optimizing outcomes and minimizing risks.

## Conclusions

This case report illustrates the successful sequential antibiotic therapy for S. maltophilia infection in a preterm neonate. Early recognition, appropriate antibiotic selection, and adjustments based on clinical and microbiological data were crucial in achieving a favorable outcome. Sequential therapy with TMP-SMX followed by levofloxacin represents an effective strategy for managing MDR S. maltophilia infections in neonates. This case underscores the importance of individualized, evidence-based approaches in managing complex neonatal infections.

## Data Availability

The original contributions presented in the study are included in the article/Supplementary Material, further inquiries can be directed to the corresponding author.
